# Why is misdiagnosis more likely among some people with rare diseases than others? Insights from a population-based cross-sectional study in China

**DOI:** 10.1186/s13023-020-01587-2

**Published:** 2020-10-28

**Authors:** Dong Dong, Roger Yat-Nork Chung, Rufina H. W. Chan, Shiwei Gong, Richard Huan Xu

**Affiliations:** 1 The Jockey Club School of Public Health and Primary Care, Faculty of Medicine, The Chinese University of Hong Kong, Shatin, Hong Kong SAR China; 2 Department of Pharmacy Business and Administration, School of Pharmacy, Tongji Medical College of Huazhong University of Science and Technology, Wuhan, Hubei China

**Keywords:** Misdiagnosis, Information accessibility, Social determinants of rare diseases, Cross-sectional survey, China

## Abstract

**Background:**

For patients with rare diseases (RD), misdiagnosis (or erroneous diagnosis) is one of the key issues that hinder RD patients’ accessibility to timely treatment. Yet, little is known about the main factors that are associated with RD patients’ misdiagnosis. The objective of this study is to analyze data from a national survey among 2040 RD patients from China to explore the association between misdiagnosis and various factors, including patients’ demographics, socio-economic status, medical history, and their accessibility to RD information.

**Results:**

Three binary logistic regression analyses were performed to assess the relationships between misdiagnosis and level of rarity of the RDs (mild, moderate, and severe), demographics, health insurance levels, and accessibility to disease-related information by using the total sample, and the adult and non-adult sub-samples. We found that accessibility to RD information is the most critical factor influencing the patients’ chances of being misdiagnosed (odds ratio [OR] = 4.459, *p* < 0.001). In other words, the greater the difficulty in accessing the information on RD management, the higher the possibility of experiencing misdiagnosis.

Such influences of information accessibility on misdiagnosis were repeatedly discovered when examining the adult (OR = 3.732, *p* < 0.001) and the non-adult (OR = 5.174, *p* < 0.001) sub-samples. The association between perceived economic status and misdiagnosis was only significant in the total sample. The only other factor significantly associated with misdiagnosis was disease multimorbidity: participants who reported no multimorbidity are less likely to experience misdiagnosis (OR = 0.42, *p* < 0.001).

**Conclusions:**

Our study indicated that patients with RDs who have difficulty in accessing disease-related information are two to five times more likely to have experienced misdiagnosis. Even after adjusting for the patients’ age, gender, economic levels, and education levels, the impact of information accessibility was still significant. Our finding highlights the importance of access to information in reducing misdiagnosis among RD patients.

## Introduction

Rare diseases (RDs) are often degenerative or even life-threatening. Currently, there are nearly 7000 RDs documented in the literature, of which 80% have genetic origins. Among patients with RDs, 50% are children, and 30% of them die before the age of five [1]. The global prevalence of RDs is approximately 10%, but the prevalence threshold and definition vary across countries. In the United States, an RD is defined as a condition that affects fewer than 2,00,000 people [2]. In Japan, the figure is 50,000 [3]. The issue of RDs has been gaining public awareness in China over the past decade. Currently, approximately 10 million Chinese are affected by one of the 7000 known RDs [4]. However, China lags far behind other countries in terms of knowledge on RDs, affecting the prevention, diagnosis treatment, and patient protection. For most RDs, there is a lack of epidemiological data at the population level and China has yet to formulate an official definition of RDs. China has so far only released its first national list of rare diseases on May 11, 2018, including 121 diseases that have relatively high prevalence and certain treatments available [5], yet without specifically defining what RDs mean in the country.

Given the low prevalence and patient base of RDs, less attention from medical and research professionals was devoted to RDs in comparison with common diseases. Thus, owing to the overall lack of knowledge surrounding these diseases, medical misdiagnosis (defined as “erroneous diagnosis” in the Chinese context) among patients with RDs is common. On average, a patient with an RD has to visit 7.3 physicians and spend 4.8 years to receive an accurate diagnosis [6]. Many, however, wait decades and some never receive one. The symptoms of RDs are often uncommon and can point in many different directions, making the diagnosis even more difficult for physicians. Graber [7] has identified a number of causes for diagnostic errors. The three most common types of errors are (1) context errors, when the diagnostic possibilities for a disease are too restrictive, (2) availability errors, when a more common or more familiar diagnosis is preferred, and (3) premature closure, which means that once a probable diagnosis is identified, other options are no longer considered [8]. As a result, a definite diagnosis and treatment are often delayed, and patients experience physical and mental discomfort and increased healthcare costs.

Previous studies have found that a wide range of socio-demographic characteristics, for example, low socioeconomic status, low educational attainment, and living in rural areas [9, 10], and clinical factors [11]; are associated with delayed diagnosis. However, there has been no relevant studies conducted to investigate misdiagnosis among RD patients in China. In this study, we conducted a preliminary population-based RD survey across China to explore the association between misdiagnosis and other factors, including patients’ demographics, socio-economic status, medical history, and their accessibility to RD information.

## Method

### Study design and participants

An online (on wenjuan.com) self-administered survey on patients’ understanding and experience of RDs was conducted in January and February 2018. The survey was approved by the Committee on the Use of Human and Animal Subjects in Teaching and Research of Hong Kong Baptist University (No: FRG2/15-16/052) and the Medical Ethics Committee of Tongji Medical College of Huazhong University of Science and Technology (No: S005).

Since there was no national registry or epidemiological studies on RD patients in China at the time of the survey, the geographical distribution and demographic characteristics of the Chinese population with RDs were unknown, making it impossible to employ probability sampling. Therefore, a non-probability, convenience snowballing method was employed to recruit participants. In collaboration with the Illness Challenge Foundation (one of the largest umbrella organizations for RD patients in China), the survey was advertised via online and offline platforms. Recruitment information was also shared by other patient organizations and individual patients to their friends and families through snowball sampling. All participants were asked to provide the names of the disease that they were diagnosed with. Those who were not able to provide the names or provided names of common diseases were excluded from the survey.

### Procedure

A brief introduction of the study and informed consent was presented to the participants on the first page of the online survey questionnaire. They had to click “Agree” to show their consent with the terms; they were also told that if they disagree, that they could simply exit the survey by closing the page. After the consent, the participants were shown to the main body of the questionnaire. At the beginning of the survey, a series of questions were used to identify the target respondents (i.e. people with RDs in China). Patients under 18 were asked to end the survey and forward the survey link to their legal guardians. Main caregivers (n = 918) and patients (n = 1089) were identified and diverted to two different versions of the questionnaire with the same measures but customized for the two groups.

### Measures

Information about patients’ demographics (year of birth, gender, hukou or household registration, current residential district, and family size), subjective socioeconomic status (measured by the respondents’ perception of their economic status in relation to others living nearby), medical history (including the year of disease onset, year of diagnosis, misdiagnosis, and the specific names of each clinically diagnosed complication), and degree of difficulty in obtaining information related to the RD were collected.

### Data analysis

Descriptive statistics were used to describe the study sample. Demographic characteristics were categorized as gender (male and female), age, hukou (dichotomized into urban and rural), and whether the participants belonged to economically developed or underdeveloped areas in China [12] (Eastern area was defined as a developed area, the other areas were defined as undeveloped areas). We also surveyed the difficulty for participants to access RD medical information (the respondent was asked to indicate how hard s/he thinks is to acquire information about his/her rare disease; scores ranged from 1 to 5, with 1 indicating ‘very easy to obtain information’ and 5 indicating ‘very hard to obtain information’), number of disease-related complications, and the participants’ family size [number of family members]). In addition, given the large income gap between developed urban area and underdeveloped rural areas in China, it was impossible to make direct comparisons based on income level. Thus, we used another item—‘perceived economic status if compared with people nearby’—as a proxy question to collect data regarding participant’s socio-economic level (scores ranged from 1 to 5, with 1 indicating ‘much lower than average’, 3 indicating ‘equal to local average’, and 5 indicating ‘much higher than average’) in the context of the place of residence. For our analysis, the perceived economic level was regrouped into three categories (below average, average, and above average).

To consider the association of rarity of disease with misdiagnosis, a total of 93 rare diseases reported by the participants were divided into three classes based on the reported prevalence of each disease: “extremely rare” with an incidence below 1/100,000, “moderately rare” with an incidence ranging from 1/100,000 to 1/10,000, and “mildly rare” with an incidence above 1/10,000. The prevalence of data was mainly obtained from Orphanet (orpha.net). Data on the prevalence of 20% of the diseases were obtained from published academic papers as their information was not available in Orphanet. The details of prevalence and data sources are listed in Additional file 1: Appendix 1.

Binary logistic regression models with the dependent variable ‘Have you been misdiagnosed?’ were then employed. This question had two response options: ‘yes’ (misdiagnosis = 1) and ‘no’ (no misdiagnosis = 0). All the models included the level of rarity, which was considered as a fixed effect. The remaining characteristics were sequentially entered into the models to assess how they were associated with the relationship between misdiagnosis and the level of rarity. Five regression models were introduced to predict the variance of such relationships in three sub-samples [general participants, adults, and non-adult (age ≤ 18 years)]. The first model directly explored the relationship between misdiagnosis and the level of rarity. The second model explored the relationship between misdiagnosis and demographic characteristics. The third model explored the relationship between misdiagnosis and RD care management. The fourth model explored the relationship between misdiagnosis and economic level, household size, as well as healthcare insurance coverage. The last model was the full model, with all the characteristics included. Fifteen models in total were presented sequentially. Moreover, the Akaike information criterion (AIC) and Bayesian information criterion (BIC) were reported to estimate the relative quality of statistical models. Analyses were performed using R (R Foundation, Austria), the statistical significance was set at *p* value < 0.05.

## Results

Table 1 displays the descriptive characteristics of the study sample. In total, 2,040 participants, with a mean age of 22.5 years, completed our survey. Among them, 53.6% were male, 52.2% registered as urban hukou, 56% came from underdeveloped areas, and more than two-thirds had experienced misdiagnosis. Descriptive statistics also revealed that nearly 20% of the participants reported having an RD within the mildly rare category, and 6.2% being extremely rare. A large proportion of the participants, 73.8%, were categorized as having a moderately rare RD.

**Table 1:** The characteristics of study sample

	Overall		Adult		Non-adult	
	N	%	N	%	N	%
Sex						
Male	1093	53.6	492	45.2	581	63.3
Female	947	46.4	597	54.8	337	36.7
Education						
No education			47	4.3	34^*^	6.7
Primary and secondary			312	28.7	172^*^	33.8
Senior			248	22.8	175^*^	34.4
College and above			482	44.3	128^*^	25.1
Disease rarity level^a^						
Mildly rare	396	19.9	86	7.8	38	4.3
Moderately rare	1466	73.8	828	75.3	638	72.0
Extremely rare	124	6.2	186	16.9	210	23.7
Hukou^b^						
Urban	1061	52.2	609	56.1	432	47.3
Rural	970	47.8	477	43.9	481	52.7
Economic developed area^c^						
Develop area	898	44.0	483	44.4	402	43.8
Underdeveloped area	1142	56.0	606	55.6	516	56.2
Have been misdiagnosed						
Yes	1310	66.1	755	72.2	534	59.1
No	671	33.9	290	27.8	370	40.9
Insurance^d^						
Free medical care			42	4.7		
Urban employee Medical insurance			359	40.4		
Urban resident medical insurance			130	14.6		
New rural cooperative medical care			357	40.2		
Difficult level to access information						
A little difficult	462	23.3	269	25.6	187	20.7
Some difficult	769	38.7	416	39.7	337	37.2
Very difficult	755	38.0	364	34.7	381	42.1
Have complication						
Yes	773	70.0	433	69.3	340	70.8
No	332	30.0	192	30.7	140	29.2
Perceived economical level as compared to locals^e^						
Below average	1342	66.9	698	64.1	644	70.2
Close to average	597	29.7	353	32.4	244	26.6
Above average	68	3.4	38	3.5	30	3.3

	Mean	SD	Mean	SD	Mean	SD
Age	22.46	17.13	36.08	10.77	6.37	4.68
Length of time to be diagnosed^f^	2.26	4.81	3.46	6.08	0.83	1.91
Family size^g^	2.73	1.14	3.27	0.87	2.08	1.08

^*^The patient’s fraternal educational level

^a^The classification of rare disease was listed in the Additional file 1: Appendix

^b^Hukou is a system of household registration in mainland China. A household registration record officially identifies a person as a resident of an area. Currently there are two categories of Hukou system: urban registration and rural registration

^c^The developed areas included Beijing, Tianjin, Hebei, Shandong, Shanghai, Jiangsu, Zhejiang, Fujian, Guangdong, Hainan, Hong Kong SAR and Macau SAR

The underdeveloped areas included Chonqing, Sichuan, Hubei, Hunan, Anhui, Jiangxi, Shaanxi, Gansu, Ningxia, Shanxi, Yunnan, Guizhou, Guangxi, Jilin, Liaoning, Heilongjiang, Inner Mongolia, Tibet, Xinjiang and Qinghai

^d^Free medical care only provides to civil servants

^e^Perceived economic level is determined by the respondent’s self-assessment of their monthly family income. It is measured by a likert scale from 1–5. If the respondents think his/her family income is about the same as average level in the places where they live, they will choose 3; whereas 1–2 means lower than average local income level, and 4–5 means higher than average level

^f^Length of time from symptom onset to an accurate diagnosis (years)

^g^Number of many family members living under the same roof

**Fig. 1:**
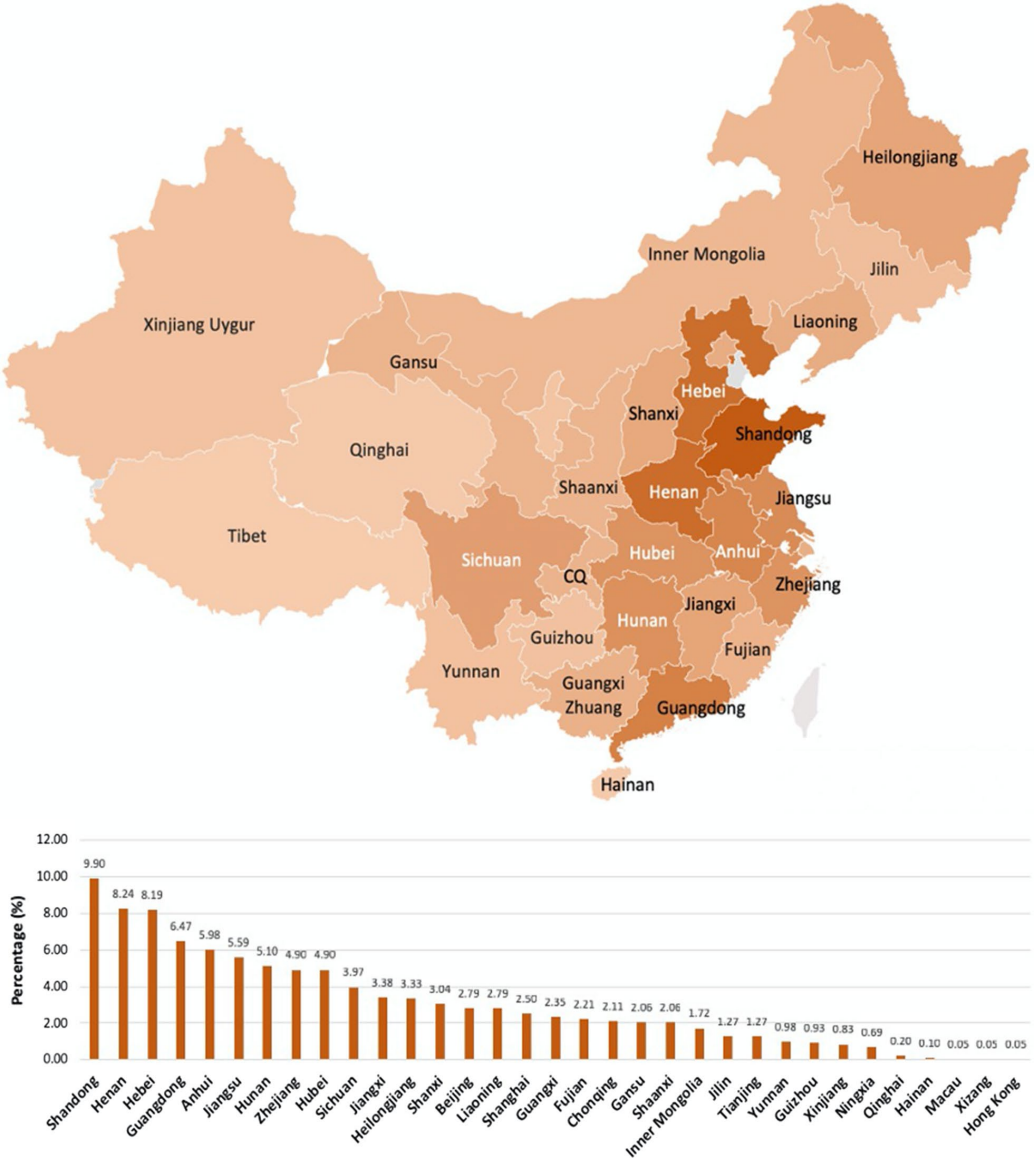
The distribution of participants reported of having rare disease in China

**Fig. 2:**
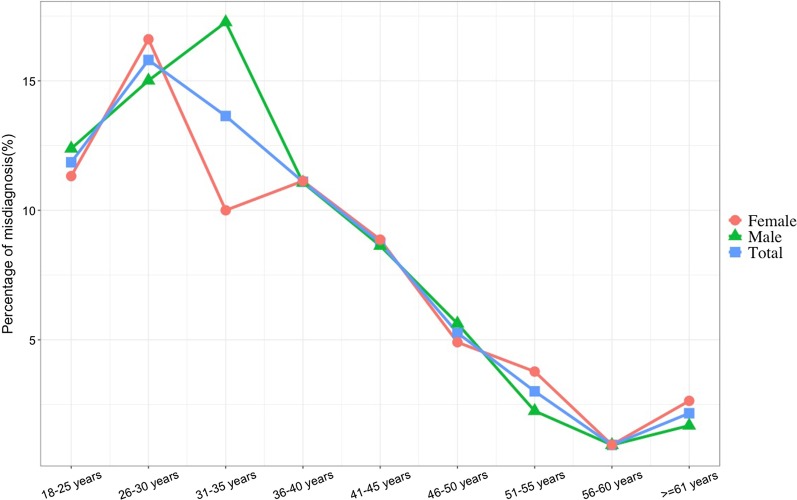
The percentage of misdiagnosis stratified by sex and age

Figure 1 presents the distribution of participants. The study sample came from all over mainland China, covering all 22 provinces, five autonomous regions, four direct-controlled municipalities, and two special administrative regions. Among them, nearly 10% came from Shandong Province, followed by Henan (8.2%) and Hebei (8.19%). The majority of the participants resided in Eastern and Southern China. For Macau, Hong Kong, and Tibet, only one participant was from each region. Overall, the highest percentage of misdiagnosis was at 26–30 years (16%) and then gradually decreased to 2% by the age of 60. Female patients who had experienced misdiagnosis were mostly aged between 26 and 30 years, and male patients aged 31–35 years old experienced the highest number of misdiagnosis when compared with other age groups (Fig. 2).

Three binary logistic regression analyses were performed to examine the relationships between misdiagnosis and level of rarity, demographics, health insurance levels, and accessibility to disease-related information by using the total sample, and the adult and non-adult sub-samples, respectively. In the total sample, gender and the level of disease rarity did not affect the likelihood of being misdiagnosed. When compared with adults, non-adults had a lower chance of being misdiagnosed (odds ratio [OR] = 0.503, 95% confidence interval (95% CI) = 0.414–0.610, *p* < 0.001). The factor of ‘difficulty in obtaining information’ revealed that the greater the difficulty in accessing information on RD management, the higher the possibility of experiencing misdiagnosis. As compared to ‘a little difficult in accessing RD information’, patients who felt ‘some difficult’ or ‘very difficult’ had a much higher probability of being misdiagnosed (OR = 2.543 for ‘some difficult’, 95% CI = 1.697–2.707, p < 0.001; OR = 3.915 for ‘very difficult’, 95% CI = 2.852- 4.732, *p* < 0.001). Moreover, participants who reported no complication are less likely to experience misdiagnosis in the full model (OR = 0.445, 95% CI = 0.335–0.590, *p* < 0.001). The economic level also had some influence on the chances of being misdiagnosed. When compared with those who perceived their family economic statuses were lower than the average local levels, those whose statuses were higher than the average were less likely to be misdiagnosed (OR = 0.778, 95% CI = 0.605–0.999, *p* < 0.05). Family size mattered too. The larger the family size, the more likely the patient are to be misdiagnosed (OR = 1.167, 95% CI = 1.040–1.230, *p* < 0.01). However, the influence of economic level and family size are not significant in the full model (Table 2).

**Table 2:** Results of logistic regression models for total participant

	Model 1^a^		Model 2		Model 3		Model 4		Model 5	
	OR	95% CI^b^	OR	95% CI	OR	95% CI	OR	95% CI	OR	95% CI
Rarity-moderate	0.764	(0.503,1.137)	0.857	(0.561,1.286)	1.162	(0.667,1.983)	0.853	(0.555,1.649)	1.329	(0.758,2.289)
Rarity-mild	0.766	(0.487,1.185)	0.901	(0.568,1.411)	0.909	(0.492,2.280)	0.839	(0.531,2.930)	1.110	(0.712,2.2)
Female			0.862	(0.721,1.067)					0.838	(0.613,1.074)
Non-adult			0.503***	**(0.414,0.610)**					0.615**	**(0.442,0.852)**
Rural Hukou			1.059	(0.875,1.287)					0.972	(0.73,1.294)
Underdeveloped area			1.130	(0.922,1.355)					1.043	(0.867,1.503)
Some difficult to access RD information					2.543***	**(1.679,2.707)**			2.709***	**(1.821,3.997)**
Very difficult to access RD information					3.915***	**(2.852,4.732)**			4.459***	**(3.283,5.615)**
No complication					0.445***	**(0.335,0.590)**			0.420***	**(0.312,0.563)**
Close to average local economic level							0.887	(0.719,1.092)	1.001	(0.756,1.411)
Above average local economic level							0.778*	**(0.605,0.999)**	1.016	(0.537,2.904)
Family size							1.167**	**(1.040,1.230)**	1.161	(0.999,1.334)

Bold value indicates significant results

Reference groups are Rarity-extremely rare, male, adult, urban hukou, developed area, a little difficult to access RD information, yes complication, below average local economic level

^a^Model 1 = level of rarity model; Model 2 = demographic model, Model 3 = Healthcare management model; Model 4 = social support model; and Model 5 = Full model

^b^*95% CI* 95% confidence interval, *OR* odds ratio

^c^*p < 0.05; **p < 0.01; ***p < 0.001

For the adult sub-sample (Table 3), while the ‘rarity of disease’ factor was non-significant in all models, ‘difficulty in obtaining RD information,’ ‘whether having multimorbidity’, and ‘type of medical insurance coverage’ are among the most significant factors associated with misdiagnosis in partial models and the full model. Patients who had difficulties accessing RD information were more likely to experience misdiagnosis (OR = 2.214 [95% CI = 1.732–4.133] and 3.496 [95% CI = 2.647–4.923], *p* < 0.001), and patients with no complication were less likely to experience misdiagnosis (OR = 0.854 [95% CI = 0.728–0.996], *p* < 0.05). When compared with patients covered by free medical insurance, the rest who were covered by urban employee insurance, urban resident insurance, or the new scheme rural insurance were all more likely to be misdiagnosed.

**Table 3:** Results of logistic regression models for adult participants

	Model 1^a^		Model 2		Model 3		Model 4		Model 5	
	OR	95% CI^b^	OR	95% CI	OR	95% CI	OR	95% CI	OR	95% CI
Rarity-moderate	0.887	(0.517,1.467)	0.956	(0.609,1.326)	0.781	(0.449,2.12)	0.981	(0.64,1.486)	0.836	(0.44,1.526)
Rarity-mild	0.854	(0.854,1.537)	0.918	(1.040,3.433)	0.751	(0.401,3.682)	0.835	(0.415,1.636)	0.809	(0.391,1.634)
Female			0.93	(0.703,1.23)					0.856	(0.619,1.179)
Age			1.001	(0.987,1.014)					0.994	(0.978,1.01)
Rural Hukou			1.275	(0.924,1.761)					1.122	(0.68,1.86)
Underdeveloped area			1.15	(0.87,1.519)					1.032	(0.744,1.428)
Primary and secondary school (9-year schooling)			1.277	(0.629,2.509)					1.515	(0.622,3.512)
High school (12-year schooling)			1.624	(0.781,3.282)					1.925	(0.764,4.647)
College and above (15-year schooling and above)			1.446	(0.706,2.869)					2.226	(0.855,5.571)
Some difficult to access RD information					2.214***	**(1.732,4.133)**			2.392***	**(1.635,3.513)**
Very difficult to access RD information					3.496***	**(2.647,4.923)**			3.732***	**(2.444,5.755)**
No complication					0.854*	**(0.728,0.996)**			0.828*	**(0.691,0.991)**
Close to average economic level							0.785	(0.635,1.254)	0.822	(0.526,1.273)
Above average economic level							0.699	(0.285,1.307)	0.824	(0.516,1.31)
Family size							1.005	(0.796,1.148)	1.056	(0.862,1.289)
Urban employee insurance							1.680	(0.853,3.376)	2.085*	**(1.017,4.22)**
Urban resident insurance							2.429*	**(1.159,4.94)**	3.097*	**(1.361,7.044)**
New scheme rural insurance							2.138*	**(1.064,4.05)**	2.987*	**(1.274,6.945)**

Bold value indicates significant results

Reference groups are Rarity-extremely rare, male, urban hukou, developed area, no education, a little difficult to access RD information, yes complication, below average local economic level, free medical insurance

Model 1 = level of rarity model; Model 2 = demographic model, Model 3 = Healthcare management model; Model 4 = social support model; and Model 5 = Full model

^a^*95% CI* 95% confidence interval, *OR* odds ratio

^b^*p < 0.05; **p < 0.01; ***p < 0.001

For the sub-sample of non-adult (Table 4), while the level of rarity did not affect the probability of misdiagnosis, age did. When the patient got 1 year older, the chances of being misdiagnosed increased by 1.113 times (95% CI = 1.059–1.171, *p* < 0.001) in model 2 and by 1.09 times in the full model (95% CI = 1.03–1.150, *p* < 0.01). Besides age, the factor of ‘difficulty in obtaining RD information’ also significantly affected the probability of being misdiagnosed in model 3 and the full model. However, none of the rest of the factors, including gender, the type of hukou, developmental status of the city of living, patients’ fraternal educational level (an important indicator of the family’s social-economic status), perceived family economic status in the local area, or family size, made a difference on misdiagnosis.

**Table 4:** Results of logistic regression models for non-adult participants (minors)

	Model 1^a^		Model 2		Model 3		Model 4		Model 5	
	OR	95% CI^b^	OR	95% CI	OR	95% CI	OR	95% CI	OR	95% CI
Rarity-moderate	0.798	(0.396,1.552)	1.535	(0.607,1.504)	1.119	(0.619,1.587)	0.801	(0.592,1.123)	2.479	(0.646,2.566)
Rarity-mild	0.932	(0.447,1.883)	1.59	(0.24,2.32)	1.298	(0.631,5.378)	0.913	(0.819,3.348)	2.63	(0.165,6.815)
Female			0.88	(0.601,1.291)					0.886	(0.485,1.573)
Age			1.113***	**(1.059,1.171)**					1.09**	**(1.03,1.150)**
Rural Hukou			1.193	(0.765,1.858)					1.076	(0.304,1.304)
Underdeveloped area			1.074	(0.734,1.576)					0.972	(0.545,1.793)
Father—primary and secondary school (9-year schooling)			0.796	(0.355,1.737)					0.878	(0.219,2.363)
Father—high school (12-year schooling)			0.834	(0.372,1.82)					0.777	(0.138,1.508)
Father—College and above (15-year schooling and above)			0.797	(0.332,1.85)					0.817	(0.1,1.483)
Some difficult to access RD information					2.549***	**(1.746,3.709)**			2.571***	**(1.535,4.516)**
Very difficult to access RD information					5.324***	**(3.579,7.739)**			5.174***	**(3.041,8.992)**
No complication					0.730***	**(0.625,0.853)**			0.735**	**(0.593,0.92)**
Close to average economic level							0.885	(0.689,1.267)	1.109	(0.512,2.043)
Above average economic level							0.888	(0.589,2.754)	1.155	(0.87,40.165)
Family size							1.028	(0.893,1.146)	0.991	(0.946,1.758)

Bold value indicates significant results

Reference groups are rarity-extremely rare, male, urban Hukou, developed area, father-no education, a little difficult to access RD information, yes complication, below average local economic level

^a^Model 1 = level of rarity model; Model 2 = demographic model, Model 3 = Healthcare management model; Model 4 = social support model; and Model 5 = Full model

^b^*95% CI* 95% confidence interval, *OR* odds ratio

^c^*p < 0.05; ** *p* < 0.01; ***p < 0.001

## Discussion

To our knowledge, this is the first study to explore factors associated with misdiagnosis of RDs based on first-hand data in China. We explored the association of misdiagnosis with patients’ demographics, socio-economic status, and healthcare factors. Our findings demonstrates that accessibility to RD information is one of the most important risk factors associated with misdiagnosis.

As opposed to conventional wisdom, whether the RD was extremely or moderately or mildly rare did not increase or decrease the probability of misdiagnosis. Perhaps only when compared with ‘common diseases’ that the level of rarity began to matter. Partly echoing to the theories on social determinants of health, we found that patients’ socio-economic characteristics might be associated with misdiagnosis. However, due to the uneven distribution of incomes and economic development in China, it was hard for us to use ‘objective standards’ (e.g., individual income, household income, etc.) to do the comparison. Therefore, we used ‘perceived economic level’—a subjective measure that gains growing popularity in studying social determinants of health [13, 14]—as an indicator of patients’ subjective assessment on their own economic status at the local level. We found that only patients who felt their economic status were higher than the average was less likely to be misdiagnosed. This is in line with the findings from previous studies which state that people with low income are more likely to lead to misdiagnosis [15, 16]. However, limited findings were reported when patients living with RDs, as seen from our data, RDs do not ‘discriminate’ in terms of gender, age, ethnicity, residential area, or educational levels.

The most important finding is that the accessibility to disease-related information seems to be the most critical factor influencing the patients’ chances of being misdiagnosed. Our models indicated that RD patients who had difficulty in accessing disease-related information are two to five times more likely to have experienced misdiagnosed, regardless of the level of rarity of their diseases. Even after adjusting for the patients' income levels and their education levels, the impact of information accessibility is still significant. This finding highlights that access to information is the key to reducing misdiagnosis. In the field of RDs, patients’ information needs are never fully met [10]. Rance et al. study shown that rare disease information sources are incompletely cross-referenced to one another and fragmented, which makes it difficult for patients to navigate across them [17].

In recent decades, the internet has become the main source for a growing number of patients with RDs as they go online to research their symptoms and obtain information about possible diseases before seeking professional help [18]. At the end of 2017, the number of netizens in China reached more than 750 million [19]. Using the internet is a cost-effective way for medical professionals, patients, and their families to obtain information about RDs [18]. However, the online sources of RDs in China are scarce; in this study, over 60% of respondents noted a general lack of available information, not to mention the questionable reliability of such information. The dominant platform for seeking and exchanging information (including experiences and knowledge) about RDs in the current studies are virtual patient communities organized based on Electronic Bulletin Boards (or BBS) or social network gadgets (such as QQ or WeChat) [20, 21]. However, such online communities are often closed and focus on one particular rare condition, which inevitably makes it hard for those with an ambiguous or unconfirmed diagnosis to join and find further help. Therefore, in order to reduce the chances of misdiagnosis, an aggregated RD information platform supported by patient communities is highly encouraged, however, previous studies also indicated concerns about the quality [10] and suitability of information [22] of such platform.

Another important source of information that can help patients manage their health is patient organizations (POs), which provide for the needs of patients with RDs. Currently, there are nearly 120 active patient groups in China, most of which are condition-specific groups, either led by patients with the same RD or initiated by physicians or medical specialists (personal communication, Yiou Wang from the Illness Challenge Foundation, 25 May 2019) [23, 24]. The support of POs is important for patients with RDs, as more than 80% of our respondents indicated a desire to join. Hall indicated that POs are the most important way to provide medical information and help patients connect with each other [25]. Moreover, Groft suggested that general support groups could provide assistance with finances and special medical equipment [26]. For patients with extremely rare diseases, POs also help disseminate useful information and provide opportunities for patients to participate in clinical trials [27]. Furthermore, in many developed countries, POs are the backbone of efforts to advocate RD management and improve public and private awareness. Overall, POs are one of the most valuable sources of patient information. Ayme et al. indicated that compared with online information, information from POs is more reliable, especially in the eyes of the patients [28].

Previous studies suggested that the most important source of information on RD management is doctor-patient communication [29–31]. Effective doctor-patient communication has the potential to help regulate patients’ emotions, facilitate comprehension of medical information, and allow for better identification of patients’ needs, preferences, and expectations [32]. However, we found that doctor-patient communication in the context of RDs might be problematic. First, adult patients living in rural areas and covered by the New Rural Cooperative Scheme reported a nearly 3.5 times higher probability of misdiagnosis compared with urban residents. This is not given the urban–rural disparity in China. Doctors from rural areas, who have less knowledge and fewer opportunities to practice with difficult cases, are incapable of providing sufficient support for patients with RDs [33, 34].

We also found that patients who reported having multimorbidity had a 44.5% higher probability of misdiagnosis; for adults, the ratio increased to 73%, and for non-adults, it increased to 85.4%. In China, high-quality healthcare resources are highly centralized at a few tertiary hospitals in big cities. A whitepaper indicated that more than 83% of Chinese doctors worked overtime [35]. Owing to time constraints, consultation times are limited, and doctors are unable to provide patients with all the necessary information. In fact, a large number of doctors complain that even they have very few means to obtain information on RDs [36]. Freitas also indicated that unmet information needs harm doctors’ decision-making, which may result in difficulties in making a definitive diagnosis [37].

In China, where there are more than 20 million people with RDs, misdiagnosis poses a huge economic and social burden on patients, families, and the healthcare system as a whole [38]. The findings of this study illustrate that improving RD patients’ ability to access disease-related information is the key in reducing misdiagnosis. In 2018, the China Alliance for Rare Diseases held its inaugural meeting in Beijing. At about the same time, the Chinese government issued the First National List of Rare Diseases. Both events demonstrate the government’s determination and ambition to manage RDs in China. It is hoped that this can serve as a starting point to a greater public and private involvement in RD management. We wish that healthcare legislations will be implemented in the next few years to provide further support for patients and researchers to study RD. We also hoped that regular awareness campaigns and local RD conferences will be held more frequently because what patients really want is to learn about living with a specific rare condition and its future impact [39].

The study has limitations. The first is the non-probability sampling strategy, which limits the generalizability of the findings. The second is the cross-sectional design, which makes it difficult to make inferences regarding causality and temporality. The third is that in asking patients to self-report the situation of their misdiagnosis, there are possibility of recall bias. The fourth is that our investigation was centered around the phenomenon of misdiagnosis in the context of RDs in general, but the associations might vary for different RDs. Finally, since the questionnaires for non-adult patients were filled by their parents, and there is a potential proxy bias.

## Conclusions

We found a very high rate of misdiagnosis of RDs across China. The difficulty in accessing disease-related information is the key cause for misdiagnosis. There were no disparities in misdiagnosis based on gender, age, geographical region, ethnicity, or education. The importance of this study lies that it is a step forward in meeting the urgent need to identify the association of patients’ socio-economic, healthcare resources, and social support characteristics with misdiagnosis of RDs. The findings can aid in the formulation of social and healthcare policy to decrease the misdiagnosis of RDs in specific target populations. The epidemic of RD misdiagnosis in China is a political emergency that needs to be urgently addressed.

## Supplementary information

**Additional file 1: Appendix: Table S1.** Rarity of each rare disease and its prevalence.

## Data Availability

The datasets used and/or analysed during the current study are available from the corresponding author on reasonable request.
