# Supplementary material for: Why is misdiagnosis more likely among some people with rare diseases than others? Insights from a population-based cross-sectional study in China
Source: Orphanet J Rare Dis. 2020 Oct 28;15:307. doi: 10.1186/s13023-020-01587-2 (PMC7594300; doi:10.1186/s13023-020-01587-2)
Supplement: Supplementary file 1 — Additional file 1: Appendix: Table S1. Rarity of each rare disease and its prevalence. [file 13023_2020_1587_MOESM1_ESM.docx]

**Additional file 1: Appendix**

**Table S1 Rarity of each rare disease and its prevalence**

| Disease | | | Prevalence | | Data source of Prevalence | |
| --- | --- | --- | --- | --- | --- | --- |
| Rarity-Severe (Prevalence < 1/100,000) | | | | | | |
|  | 1q44 microdeletion syndrome | <1 /1,000,000 | | orpha.net | |  |
|  | Acute transverse myelitis | 1/1,000,000 ~ 1/250,000 | | orpha.net | |  |
|  | Alexander disease | 1/2,700,000 | | orpha.net | |  |
|  | Alpers Huttenlocher syndrome | <1 /1,000,000 | | orpha.net | |  |
|  | Alstrom Syndrome | 1-9 /1,000,000 | | orpha.net | |  |
|  | atypical haemolytic uraemic syndrome,aHUS | 1-9 / 1 000 000 | | orpha.net | |  |
|  | Bartter syndrome | 1/1 000 000 | | orpha.net | |  |
|  | Chediak-Higashi syndrome | 500 cases reported | | orpha.net | |  |
|  | Erythrokeratoderma | 200 cases reported | | orpha.net | |  |
|  | GM1 gangliosidosis | 1/100,000-1/200,000 in live births | | orpha.net | |  |
|  | Growth hormone deficiency | 1-9 /1,000,000 | | Stanley T. (2012). Diagnosis of growth hormone deficiency in childhood. Current opinion in endocrinology, diabetes, and obesity, 19(1), 47–52. doi:10.1097/MED.0b013e32834ec952 | |  |
|  | Jacobsen Syndrome | 200 cases reported | | orpha.net | |  |
|  | Lymphangioleio-myomatosis | 1-9 /1,000,000 | | orpha.net | |  |
|  | Massive osteolysis/Gorham-Stout disease | 300 cases reported | | orpha.net | |  |
|  | Metachromatic leukodystrophy | 1-9 /1,000,000 | | orpha.net | |  |
|  | Mitochondrial encephalopathy | 1-9 / 1,000,000 | | orpha.net | |  |
|  | Niemann-Pick disease | <1 /1,000,000 | | orpha.net | |  |
|  | Peutz–Jeghers syndrome | 1-9 /1,000,000 | | orpha.net | |  |
|  | Progressive diaphysial dysplasia/Camurati-Engelmann disease | 300 cases reported | | orpha.net | |  |
|  | Spondyloepiphyseal Dysplasia Congenita | 1 per 100,000 live births | | orpha.net | |  |
|  | Triple-A syndrome (Allgrove syndrome) | <1 /1,000,000 | | orpha.net | |  |
| Rarity- Moderate (1/10,000<Prevalence < 1/100,000) | | | | | | |
|  | Achondroplasia | 1-9 / 100,000 | | orpha.net | |  |
|  | Achromatopsia | 1-9 / 100,000 | | orpha.net | |  |
|  | Acromegaly | 1-9 / 100,000 | | orpha.net | |  |
|  | Adrenal Hypoplasia Congenita | less than 1/12,500 births | | AvRuskin, T., Krishnan, N., & Juan, C. (2004). Congenital Adrenal Hypoplasia and Male Pseudohermaphroditism Due to DAX1 Mutation, SF1 Mutation or Neither: A Patient Report. Journal of Pediatric Endocrinology and Metabolism, 17(8), 1125-1132. | |  |
|  | Albinism | 1/10,000-1/20,000 | | Mártinez‐García, M. and Montoliu, L. (2013), Albinism in Europe. J Dermatol, 40: 319-324. doi:10.1111/1346-8138.12170 | |  |
|  | Amyotrophic lateral sclerosis | 1-9 / 100,000 | | orpha.net | |  |
|  | Angelman syndrome | 1-9 / 100,000 | | orpha.net | |  |
|  | Anti-neutrophil cytoplasmic antibody-associated vasculitis | 4.6-18.4/100,000 | | Watts, R., Mahr, A., Mohammad, A., Gatenby, P., Basu, N., & Flores-Suárez, L. (2015). Classification, epidemiology and clinical subgrouping of antineutrophil cytoplasmic antibody (ANCA)-associated vasculitis. Nephrology Dialysis Transplantation, 30(Suppl1), I14-I22. | |  |
|  | Behcet disease | 1-9/100,000 | | orpha.net | |  |
|  | Cerebral palsy | 1.5-4/1000 in live births | | Stavsky, M., Mor, O., Mastrolia, S., Greenbaum, S., Than, N., & Erez, O. (2017). Cerebral Palsy-Trends in Epidemiology and Recent Development in Prenatal Mechanisms of Disease, Treatment, and Prevention. Frontiers in Pediatrics, 5, 21. | |  |
|  | Citrullinemia | 1-9 /100,000 | | orpha.net | |  |
|  | Congenital Adrenal Hyperplasia | 1-9 / 100,000 | | orpha.net | |  |
|  | Cri-du-chat syndrome | 1/20 000-1/50 000 newborns | | orpha.net | |  |
|  | Crohn's diseas | 1.2-21.2/100,000 | | Prideaux, L., Kamm, M. A., De Cruz, P. P., Chan, F. K., & Ng, S. C. (2012). Inflammatory bowel disease in Asia: a systematic review. Journal of gastroenterology and hepatology, 27(8), 1266-1280. | |  |
|  | De Lange syndrome | 1-9 / 100,000 | | orpha.net | |  |
|  | Duchenne Muscular Dystrophy | 1-9 / 100,000 | | orpha.net | |  |
|  | Eisenmenger's syndrome | 1-9 / 1 000 000 | | orpha.net | |  |
|  | Epidermolysis bullosa | 1-9 /1,000,000 | | orpha.net | |  |
|  | Fabry disease | 1 / 100,000 | | Branton, M. H., Schiffmann, R., Sabnis, etc. (2002). Natural history of Fabry renal disease: influence of α-galactosidase A activity and genetic mutations on clinical course. Medicine, 81(2), 122-138. | |  |
|  | Gaucher disease | 1-9 / 100,000 | | orpha.net | |  |
|  | Glycogen storage disease due to acid maltase deficiency | 1-9 / 100,000 | | orpha.net | |  |
|  | Glycogen storage disease II | 1-9 / 100,000 | | orpha.net | |  |
|  | Granulomatosis with Polyangiitis | 1-9 / 100,000 | | orpha.net | |  |
|  | Hemolytic anemia due to red cell pyruvate kinase deficiency | 1-9 / 100,000 | | orpha.net | |  |
|  | Hemophilia | 1-9 / 100,000 | | orpha.net | |  |
|  | Hepatolenticular degeneration/Wilson disease | 1-9 / 100,000 | | orpha.net | |  |
|  | Huntington's disease | 1-9 / 100,000 | | orpha.net | |  |
|  | Ichthyosis | average of subtypes | | orpha.net | |  |
|  | Idiopathic Hypogonadotropic Hypogonadism | 1/4000-1/10,000 in males, and 2 to 5 times less frequent in females | | Silveira, L. G., & Latronico, A. C. (2013). Approach to the Patient with Hypogonadotropic Hypogonadism. The Journal of Clinical Endocrinology & Metabolism, 98(5), 1781-1788. | |  |
|  | Immunologic thrombocytopenic purpura | 5/100,000 | | Fogarty, P. F., & Segal, J. B. (2007). The epidemiology of immune thrombocytopenic purpura. Current opinion in hematology, 14(5), 515-519. | |  |
|  | Kallmann Syndrome,KS | 1-9 / 100,000 | | orpha.net | |  |
|  | Mucolipidosis type IV | 1/40 000 births | | orpha.net | |  |
|  | Mucopolysaccharidosis | 1-9 / 100,000 | | orpha.net | |  |
|  | Multiple Sclerosis | 1-2/100,000 | | Cheng, Q, Cheng, X-J, & Jiang, G-X. (2009). Multiple sclerosis in China—history and future. Multiple Sclerosis, 15(6), 655-660. | |  |
|  | Myasthenia Gravis | 1-9 / 100,000 | | orpha.net | |  |
|  | Myelodysplastic syndromes | 1-9 / 100,000 | | orpha.net | |  |
|  | Neuromyelitis optica | 1-9 / 100,000 | | orpha.net | |  |
|  | Noonan syndrome | 1/1,000-1/2500 live births | | orpha.net | |  |
|  | Ornithine transcarbamylase deficiency | 1-9 / 100,000 | | orpha.net | |  |
|  | Prader-Willi syndrome | 1-9 / 100,000 | | orpha.net | |  |
|  | Pseudoachondroplasia | 1-9 / 100,000 | | orpha.net | |  |
|  | Pseudomyxoma peritonei | 1-9 / 100,000 | | orpha.net | |  |
|  | Pulmonary hypertension | 1-9 / 100,000 | | orpha.net | |  |
|  | Retinoblastoma, Rb | 1/20,000~1/15,000 | | orpha.net | |  |
|  | Sclerosis | 5/100,000 | | Tullman, M. (2013). Overview of the epidemiology, diagnosis, and disease progression associated with multiple sclerosis. The American Journal of Managed Care, 19(2 Suppl), S15-S20. | |  |
|  | Spinal cord tumor | 0.7-3.6/100 000 | | Newton, H. B. (2016). Handbook of Neuro-Oncology Neuroimaging: Second Edition. Elsevier: 35-39. | |  |
|  | Spinal muscular atrophy | 1-9 / 100,000 | | orpha.net | |  |
|  | Spinocerebellar ataxias | 1-9 / 100,000 | | orpha.net | |  |
|  | Systemic Vasculitis | 1-2/100 000 | | Lane, S., Watts, E., & Scott, R. (2005). Epidemiology of systemic vasculitis. Current Rheumatology Reports, 7(4), 270-275. | |  |
|  | Takayasu arteritis | 1-9 / 100,000 | | orpha.net | |  |
| Rarity - Mild (Prevalence >1/10,000) | | | | | | |
|  | Charcot-Marie-Tooth disease | 1-5 / 10,000 | | orpha.net | |  |
|  | Fuchs' syndrome | 3.7–9.2% in patients over 50 years of age | | Pilger, Daniel, Brockmann, Claudia, Maier, Anna-Karina B., & Bertelmann, Eckart. (2019). Predictive Factors for Clinical Outcomes after Primary Descemet's Membrane Endothelial Keratoplasty for Fuchs' Endothelial Dystrophy. Current Eye Research, 44(2), 147-153. | |  |
|  | Hereditary hemorrhagic telangiectasia | 1-5 / 10,000 | | orpha.net | |  |
|  | hyperammonemia | 1-5 / 10,000 | | orpha.net | |  |
|  | Hypopituitarism | 4.5/1,0000 | | Aimaretti G, Kreitschmann-Andermahr I, Stalla GK, Ghigo E ( 2007). Hypopituitarism. Lancet. 369 (9571): 1461–70. | |  |
|  | Isolated spina bifida | 1-5 / 10 000 | | orpha.net | |  |
|  | Keratoconus | 5.4/1,0000 | | Gokhale N. S. (2013). Epidemiology of keratoconus. Indian journal of ophthalmology, 61(8), 382–383. doi:10.4103/0301-4738.116054 | |  |
|  | Klinefelter syndrome | 1/1,000 | | Wattendorf DJ, Muenke M. （2005） Klinefelter syndrome.Am Fam Physician. 72(11):2259-62. | |  |
|  | Marfan Syndrome | 1-5 / 10,000 | | orpha.net | |  |
|  | Mitochondrial disorders | at least 1/5000 | | Schaefer, A., Taylor, R., Turnbull, D., & Chinnery, P. (2004). The epidemiology of mitochondrial disorders—past, present and future. BBA - Bioenergetics, 1659(2-3), 115-120. | |  |
|  | Neurofibromatosis | 1-5 / 10,000 | | orpha.net | |  |
|  | Noncompaction of the ventricular myocardium | 1.4/1,0000 | | Vizzardi, E., Nodari, S., Metra, M., & Dei Cas, L. (2006). Non-compaction of the ventricular myocardium. Heart international, 2(3-4), 178. doi:10.4081/hi.2006.178 | |  |
|  | Osteogenesis imperfecta | 1-5 / 10,000 | | orpha.net | |  |
|  | Pemphigus vulgaris | 1-5 / 10,000 | | orpha.net | |  |
|  | Primary adrenal insufficiency/Addison's disease | 1-5 / 10,000 | | orpha.net | |  |
|  | Retinitis Pigmentosa, RP | 1-5 / 10,000 | | orpha.net | |  |
|  | Stargardt disease | 1-5 / 10,000 | | orpha.net | |  |
|  | Systemic lupus erythematosus | 1-5 / 10,000 | | orpha.net | |  |
|  | Systemic sclerosis | 1-5 / 10,000 | | orpha.net | |  |
|  | Tuberous sclerosis complex | 1-5 / 10,000 | | orpha.net | |  |
|  | Turner syndrome | 1-5 / 10,000 | | orpha.net | |  |
|  | Uveitis | 1-5 / 10,000 | | orpha.net | |  |
|  | Charcot-Marie-Tooth disease | 1-5 / 10,000 | | orpha.net | |  |
